# Stalking of Psychiatrists by Patients: A Literature Review on Early Warning Signs, Prevention Strategies, and Forensic Implications

**DOI:** 10.1192/j.eurpsy.2026.11284

**Published:** 2026-07-31

**Authors:** Y. Imbert, H. Raai

**Affiliations:** Behavioral Health, SBH Health System, New York, United States

## Abstract

**Introduction:**

Psychiatrists face a unique occupational risk when patients engage in repeated, unwanted behaviors defined clinically as stalking leading to fear, distress, and potential harm. Early detection and preventive measures are critical, yet formal training and institutional protocols on these matters are limited. In the present case report, we present the case of a male patient with a history of Bipolar I Disorder who exhibited stalking behaviors toward a former psychiatrist through repeated social media contact and phone calls to the psychiatrist’s parents. Using this case as an example, we will discuss early warning signs, preventive strategies, and ways to detect high-risk behaviors in such patients.

**Objectives:**

To review available literature on stalking of psychiatrists by patients, with emphasis on identifying early warning signs, outlining prevention strategies, and understanding the high-risk behaviors in patients.

**Methods:**

A literature review was conducted, focusing on peer-reviewed articles, commentary pieces, and professional guidelines published between 2000 and 2024. Keywords included “stalking by patients,” “psychiatrists,” “early warning signs,” “prevention,” and “forensic risk.”

**Results:**

Prevalence and Risk Factors

Up to 20% of psychiatrists report having been stalked by a patient at least once in their careers².

Patients who stalk often have psychotic or personality disorders—particularly Cluster B—and may exhibit erotomanic delusions or fixation syndromes².

Early Warning Signs

Unsolicited contact via telephone, email, letters, or social media³.

Boundary violations¹.

Attempts to involve third parties, such as contacting family members or colleagues¹.

Prevention Strategies

Individual Level

Establish professional boundaries from the outset³.

Limit personal information online³.

Document all boundary-testing incidents contemporaneously³.

Clinical and Institutional Level

Provide staff training on recognition of stalking behaviors and response protocols³.

Develop and disseminate policies for reporting and managing stalking incidents, including escalation pathways to legal counsel and security services³.

High-Risk Behaviors

Stalking may escalate to threats, property damage, or physical assault, necessitating involvement of law enforcement¹.

Detailed risk assessments and safety planning for clinicians and staff are essential³.

**Conclusions:**

Stalking by patients represents a serious, underrecognized risk in psychiatric practice. Early identification of warning signs, reinforced by individual and institutional preventive strategies, can help mitigate harm. Awareness of potential forensic implications ensures that psychiatrists can balance therapeutic responsibilities with personal safety. Enhanced training, clear policies, and multidisciplinary collaboration are recommended to address this occupational hazard.

**Disclosure of Interest:**

None Declared

